# Network pharmacology and molecular docking reveal the mechanisms of curcumin activity against esophageal squamous cell carcinoma

**DOI:** 10.3389/fphar.2024.1282361

**Published:** 2024-04-03

**Authors:** Jian Wang, Zhilong Zhang, Qian Li, Zilong Hu, Yuan Chen, Hao Chen, Wei Cai, Qiancheng Du, Peng Zhang, Dian Xiong, Shugao Ye

**Affiliations:** ^1^ Department of Thoracic Surgery, Shanghai Xuhui Central Hospital, Shanghai, China; ^2^ Department of General Practice, The Affiliated Wuxi People’s Hospital of Nanjing Medical University, Wuxi, China; ^3^ Fuzhou Medical College of Nanchang University, Fuzhou, Jiangxi, China; ^4^ Lung Transplantation Center, Department of Thoracic Surgery, The Affiliated Wuxi People’s Hospital of Nanjing Medical University, Wuxi, China; ^5^ Department of Cardiothoracic Surgery, The Second Affiliated Hospital of Nanchang University, , Nanchang, Jiangxi, China

**Keywords:** cell cycle arrest, curcumin, esophageal squamous cell carcinoma, molecular docking, network pharmacology

## Abstract

**Background:** Curcumin (CUR), an effective traditional Chinese medicinal extract, displays good anti-cancer activity against various cancers. Nevertheless, the impacts and fundamental mechanisms of CUR to treat esophageal squamous cell carcinoma (ESCC) yet to be comprehensively clarified. This study examined the suppressive impacts of CUR on ESCC.

**Methods:** For a comprehensive understanding of the effect of CUR in ESCC. The CUR targets and ESCC-related genes were identified respectively, and the intersection targets between CUR and ESCC were acquired. Then, we examined the intersection targets and discovered genes that were expressed differently in ESCC. Using DAVID, enrichment analyses were conducted on the targets of CUR-ESCC. The STRING database and Cytoscape v.3.9.1 were utilized to build networks for protein-protein interaction (PPI) and drug-target-pathway. Furthermore, the interactions between CUR and its core targets were confirmed by molecular docking studies. To confirm the effects of CUR on ESCC cells, *in vitro* experiments were finally conducted.

**Results:** Overall, 47 potential CUR targets for ESCC treatment were identified. The KEGG pathway enrichment analysis identified 61 signaling pathways, primarily associated with the FoxO signaling, the cell cycle, cellular senescence, the IL-17 signaling pathway which play important roles in ESCC progression. In the PPI network and the docking results identified CHEK1 and CDK6 as the core targets that positively associated with ESCC survival. CUR arrested ESCC cells at the G2/M and S phases, as shown by flow cytometry. Colony formation and CCK8 assays showed that CUR can inhibit the proliferative ability of ESCC cells. The Transwell invasion results validated that CUR can significantly inhibit the invasion rates of ESCC cells.

**Conclusion:** Collectively, these findings indicate that CUR exhibits pharmacological effects on multiple targets and pathways in ESCC.

## 1 Introduction

Esophageal cancer (EC) is one of the major contributors to cancer-related fatalities globally (Siegel et al., 2023). Around 80% of ECs worldwide are squamous cell carcinomas, which account for the predominant histological subtype (Colquhoun et al., 2015; Abnet et al., 2018). Despite the progress made in systemic treatment, radiation therapy, and surgical procedures, cancers such as ESCC continue to be difficult to manage, resulting in a significantly low survival rate of only 5% after 5 years (Morgan et al., 2022). Furthermore, systemic chemotherapy for ESCC has several drawbacks, including considerable side effects and drug resistance. Plant-derived natural products are important sources of low-toxicity anti-cancer agents and high efficacy has been observed from plant-derived natural products (Gordaliza, 2007). For example, plant-based substances are significant reservoirs of anti-cancer compounds that have minimal toxicity and exhibit remarkable effectiveness, including ESCC (Imai et al., 2016; Ai et al., 2022). In recent years, traditional Chinese medicines (TCM) have been extensively studied for their ability to treat ESCC (Ying et al., 2018; Li et al., 2022).

Curcumin (CUR), an effective compound found in turmeric (Curcuma longa), has been applied in TCM and as a culinary ingredient since ancient times (Li et al., 2020). Numerous lines of evidence indicate that CUR has a potential in the management of various forms of cancer, including prostate cancer (Termini et al., 2020), colorectal cancer (Pricci et al., 2020), breast cancer (Deng et al., 2022), endometrial carcinoma (Zhang et al., 2019) and non-small cell lung cancer (Xie et al., 2022). CUR has potential anti-ESCC activity. For instance, CUR can induce apoptosis and enhance the sensitivity of fluorouracil ESCC cell death by suppressing the NF-κB signaling pathway (Tian et al., 2012). According to Deng and his colleagues, the combination of CUR and docetaxel triggers cell death and self-degradation in ESCC cells through the PI3K/AKT/mTOR signaling pathway (Deng et al., 2021). In ESCC cells, CUR inhibits STAT3-mediated signaling and induces apoptosis and growth arrest (Liu et al., 2018). A preclinical study using an orthotopic tumor xenograft model found that a novel CUR analog (SSC-5) inhibited ESCC growth and invasion (Tung et al., 2018). However, CUR has not yet been fully elucidated as a cancer-fighting agent in ESCC.

The treatment of diseases with TCM involves targeting multiple targets and pathways. Therefore, big data can be used to identify potential signaling pathways and existing targets of CUR and ESCC. Network pharmacology is a comprehensive method that combines bioinformatics and pharmacology. By combining data and computational analysis, this approach has the ability to establish a structured connection between drugs and diseases, enabling a better comprehension of drug mechanisms (Wei et al., 2021).

In this study, we used network pharmacology and molecular coupling techniques to screen and predict potential targets of CUR and CUR activated signaling pathways for the treatment of ESCC and provided scientific evidence for the development and application of these drugs. This study provides novel insights for the treatment of ESCC.

## 2 Materials and methods

### 2.1 Collection of CUR-related targets and ESCC-related genes

Table 1 lists the databases used in this study. The PubChem database provided the 2D chemical structure and the ‘SMILES’ format of CUR. Access to the Swiss target prediction database was made by using the CUR compound in the ‘SMILES’ format. In addition, the Pharmapper database was used to obtain the CUR targets for a comprehensive collection of drug targets. ESCC-related genes were identified in the GeneCards database using the phrase “esophageal squamous cell carcinoma” as the keyword. The OMIM and TTD databases were also used. The UniProt database was used to convert all targets into standardized gene symbols. Finally, duplicate and nonstandard targets of CUR and ESCC were eliminated. Venny v.2.1.0 was used to identify potential CUR targets relevant to ESCC by intersecting them with ESCC-related genes. From TCGA data set (http://portal.gdc.com), we downloaded the expression profiles of RNA sequencing (level 3) and clinical information of patients with ESCC. To increase the credibility of potential CUR targets against ESCC, we specifically chose the genes that exhibited differential expression in ESCC within the TCGA dataset.

**TABLE 1:** Basic information of the database used for the screening of curcumin in the treatment of esophageal squamous cell carcinoma.

Name	URL
PubChem	https://pubchem.ncbi.nlm.nih.gov/
PharmMapper	http://lilab-ecust.cn/pharmmapper/
SwissTargetPrediction	http://www.swisstargetprediction.ch/
GeneCards	https://www.genecards.org/
OMIM	https://www.omim.org/
TTD	https://db.idrblab.net/ttd/
Venny 2.1.0	https://bioinfogp.cnb.csic.es/tools/venny/index.html
TCGA	https://www.cancer.gov/ccg/research/genome-sequencing/tcga
DAVID	https://david.ncifcrf.gov/
STRING	https://cn.string-db.org/
Uniprot	https://www.uniprot.org/
Bioinformatics	http://www.bioinformatics.com.cn/
KEGG Mapper	https://www.kegg.jp/kegg/mapper/

### 2.2 GO and KEGG enrichment analysis

Gene Ontology (GO) and Kyoto Encyclopedia of Genes and Genomes (KEGG) pathway enrichment of commonly selected targets of CUR and ESCC were analyzed using DAVID. We selected the top 20 GO items and KEGG pathways respectively and visualized them on a bioinformatics website.

### 2.3 Screening of network building and core targets

We applied previously reported methodologies (Xiong et al., 2023) to construct a PPI and drug target pathways. In Cytoscape v.3.9.1, the maximum clique centralities (MCC), degrees, maximum neighborhood components (MNC), and closeness were utilized to filter the top 15 targets. The four calculation methods determined the core targets based on their intersections.

### 2.4 Molecular docking

The procedure for docking small molecules to proteins has been described previously (Xiong et al., 2023). In brief, PubChem (https://pubchem.ncbi.nlm.nih.gov/) provided the SDF format file of the three-dimensional structure of CUR. In ChemDraw (http://www.perkinelmer.com/tw/category/chemdraw), the small molecule of CUR was imported. Protein Data Bank (http://www.rcsb.org/) was used to download the initial structures of the top 11 potential targets, and were visualized using PyMoL. AutoDock MGLTools 1.5.6 was used to hydrogenate, calculate the charge, and calculate the non-polar hydrogen combination of the target proteins. PDBQT was used to store the results. Lastly, we used AutoDock Vina 1.1.2 to simulate molecular docking, and PyMOL to visualize the results.

### 2.5 Survival analysis

Based on TCGA datasets, RNA sequencing counts (level 3) and clinical information for patients with ESCC were obtained. Guidelines and policies were followed during acquisition and application. Additionally, Survival differences between the two groups were compared using Kaplan-Meier (KM) analysis. KM curves were analyzed using log-rank tests and univariate Cox proportional hazards regression to generate *p*-values and risk ratios with 95% confidence intervals (CIs). R software v.4.0.3 was used for all analytical methods and application of R packages.

### 2.6 Cell culture and treatment

The Chinese Academy of Sciences provided KYSE-140, which is one of the cell lines for human ESCC. KYSE-140 was cultured in RPMI 1640 (HyClone, Logan, UT, United States) with supplements of 10% FBS (Gibco, Waltham, MA, United States) at 37 °C in 5% CO_2_/95% air.

### 2.7 Flow cytometry

KYSE-140 cells were treated with diluted CUR (Yuanye Bio-Technology, Shanghai, China) at 20 μΜ and 40 μΜ. The negative control (NC) group was cultured in drug-free medium. After 48 h, the PI staining (Fcmacs, Nanjing, China) was performed on the cells, and the flow cytometry (BD FACS Aria III) was used to detect the cell cycle.

### 2.8 Cell Counting Kit-8 (CCK-8) assay and colony formation

The Cell Counting Kit-8 (CCK-8) was obtained from Yesen (Shanghai, China). The methodologies used for CCK-8 and colony formation assays have been described previously (Xiong et al., 2018).

### 2.9 Transwell assay

Cells (1 × 10^5^) were suspended in serum-free medium supplemented with specific concentrations of CUR or in drug-free medium. The cells were placed in the top section of pre-coated wells that already had 50 μL of diluted Matrigel. (1:6; BD Biosciences, San Jose, CA, United States). In the lower compartments, there was a presence of 1,640 medium enriched with 30% FBS. The culture was conducted for 48 h at a temperature of 37 °C in a thermostatic incubator with 5% CO_2_. Finally, inverted microscopy was used to count cells (stained with 0.5% crystal violet) attached to the underside of the Transwell chambers.

### 2.10 Western blot analysis

Western blot analysis has been described in previously (Xiong et al., 2023). Primary antibodies: rabbit antibody against CDK2 (10122-1-AP, 1:1,000 dilution; ProteinTech), rabbit antibody against RB (17218-1-AP, 1:800 dilution; ProteinTech), rabbit antibody against p-RB (30376-1-AP, 1:2000 dilution; ProteinTech).

### 2.11 Measurement of senescence-associated β-galactosidase (SA-β-gal)

SA-β-gal has been described in previously (Xiong et al., 2023).

### 2.12 Statistical analysis

GraphPad Prism v.9.0 was used for statistical analysis. The mean and standard deviation (SD) are used to express the data. For the purpose of comparing two or more groups, we conducted a one-way analysis of variance (ANOVA). Additionally, for comparing two groups, we conducted either a two-tailed Student’s t-test or a Wilcoxon test. Statistical significance was set at *p*-values <0.05.

## 3 Results

### 3.1 Targets of CUR and CC

**FIGURE 1:**
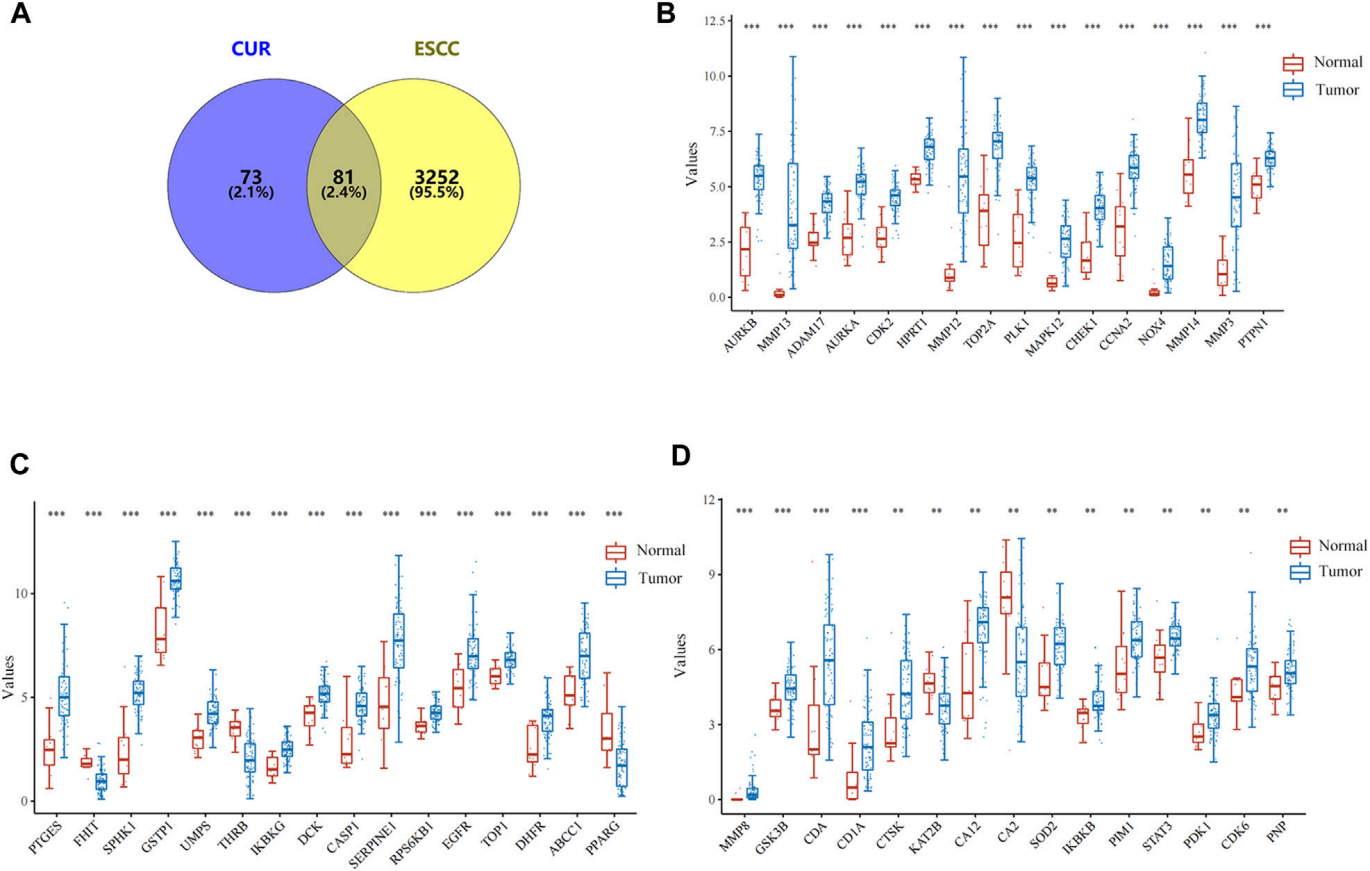
Targets of curcumin (CUR) relevant to the treatment of esophageal squamous cell carcinoma (ESCC). **(A)** Venn diagram showing the intersection of CUR and ESCC targets. **(B–D)** The expression distribution of genes in ESCC tissues and normal tissues. *p < 0.05, **p < 0.01, ***p < 0.001.

In total, 154 CUR targets were obtained from the SwissTargetPrediction and Pharmapper databases. In total, there were 3333 ESCC-related genes were acquired from the GeneCards, OMIM, and TTD databases. Using Venny v.2.1.0, we identified 81 potential CUR targets of ESCC (Figure 1A). The expression distribution of 81 genes in ESCC and normal esophageal tissues was then analyzed using TCGA data set. A total of 47/81(58.02%) mRNA transcripts were differentially expressed in TCGA dataset (Figures 1B–D).

### 3.2 GO and KEGG enrichment analysis revealed the possible signaling pathway by which CUR played an anti- ESCC role

**FIGURE 2:**
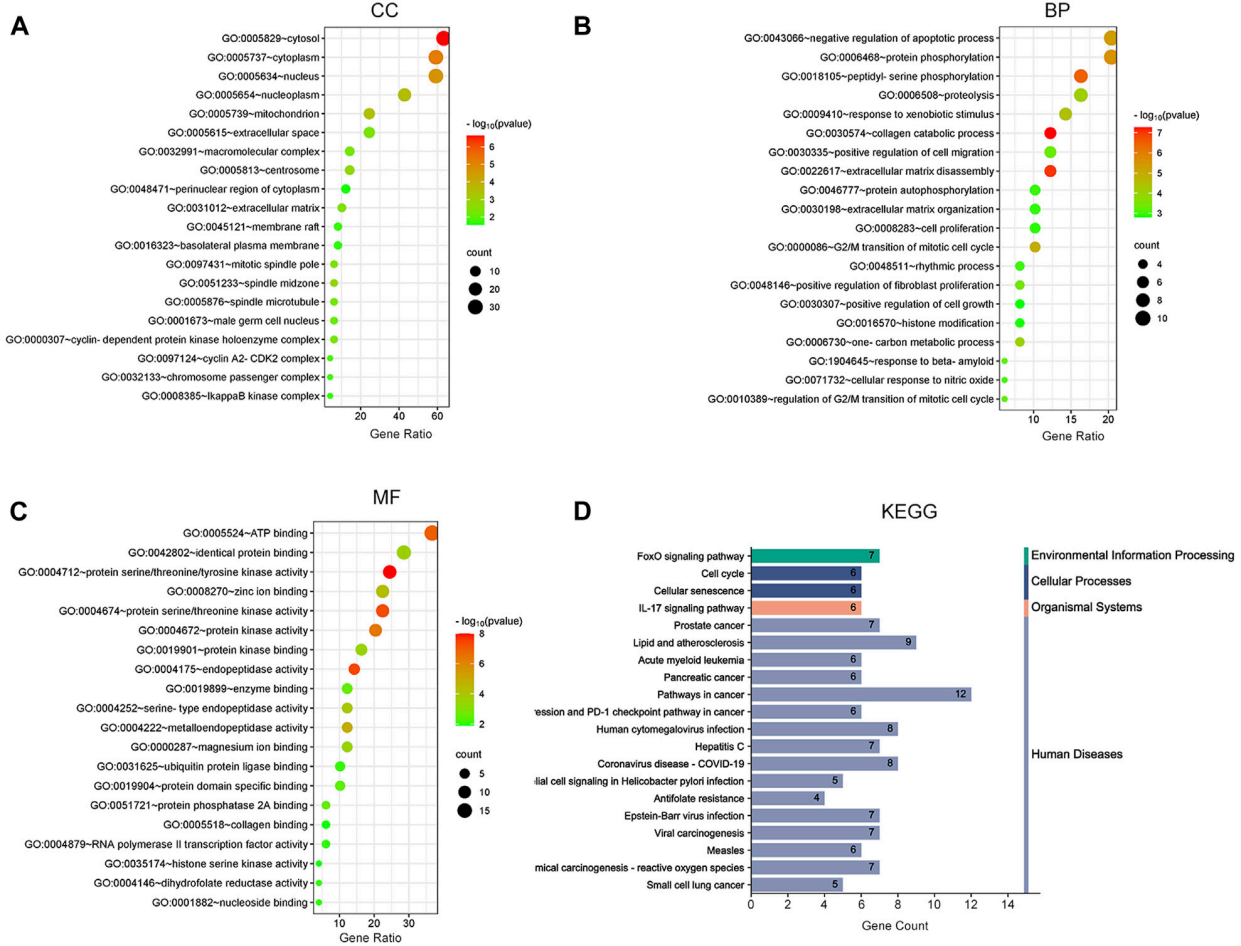
Bubble plot of enrichment analysis. **(A–C)** GO functional enrichment analysis of curcumin (CUR) in esophageal squamous cell carcinoma (ESCC). **(D)** KEGG pathway enrichment analysis of CUR in treating ESCC.

**FIGURE 3:**
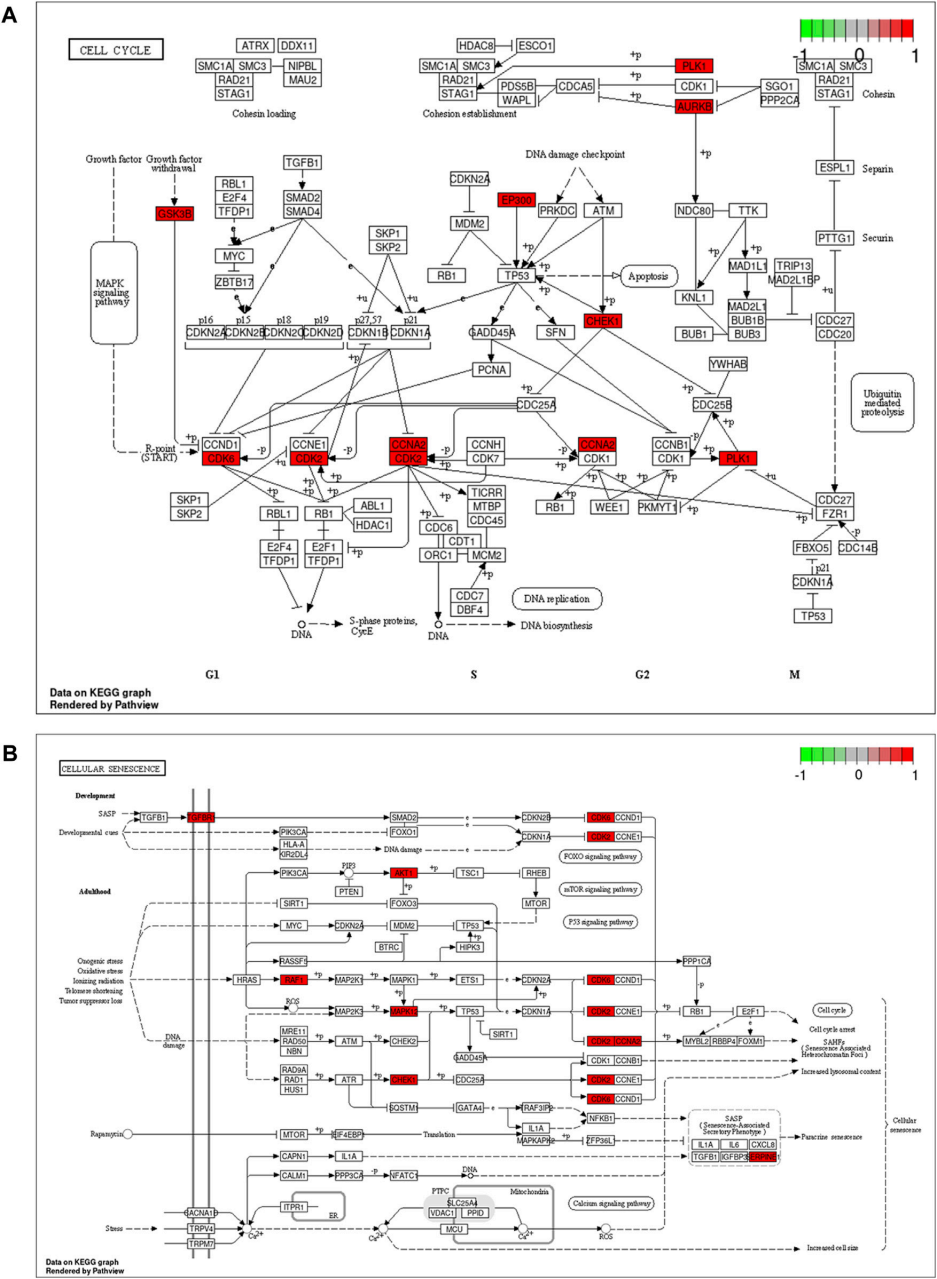
Cell cycle signaling pathway **(A)** and cellular senescence **(B)**. Red marks represent potential targets for curcumin (CUR) intervention.

To demonstrate the anti-ESCC mechanisms of CUR, we conducted GO and KEGG pathway enrichment analyses on the 47 genes that showed differential expression. GO and KEGG pathways enriched by DAVID identified 47 potential CUR targets to treat ESCC. There were 152 entries in the GO enrichment analysis (*p* < 0.05), including BP, 89; CC, 26; and MF, 37. For the bioinformatics analysis, the most significant 20 entries for each type of analysis were chosen and displayed visually (Figures 2A–C). BPs primarily included the collagen catabolic process, extracellular matrix disassembly, protein phosphorylation, negative regulation of the apoptotic process, G2/M transition of the mitotic cell cycle, and mainly involved the cytosol, cytoplasm, nucleus, nucleoplasm and mitochondrion. MFs mainly involved in protein serine/threonine/tyrosine kinase activity, endopeptidase activity, protein serine/threonine kinase activity and protein kinase activity. The KEGG pathway analysis enriched 61 signaling pathways (*p* < 0.05) and the most significant 20 pathways were chosen and displayed visually (Figure 2D). The main pathways involved were the FoxO signaling pathway, cell cycle, cellular senescence, IL-17 signaling pathway, and cancer pathways. Among them, the cell cycle and cellular senescence pathways were the most important and were selected for mapping (Figure 3).

### 3.3 PPI network and drug-target-pathway network identify potential CUR targets for ESCC treatment

**FIGURE 4:**
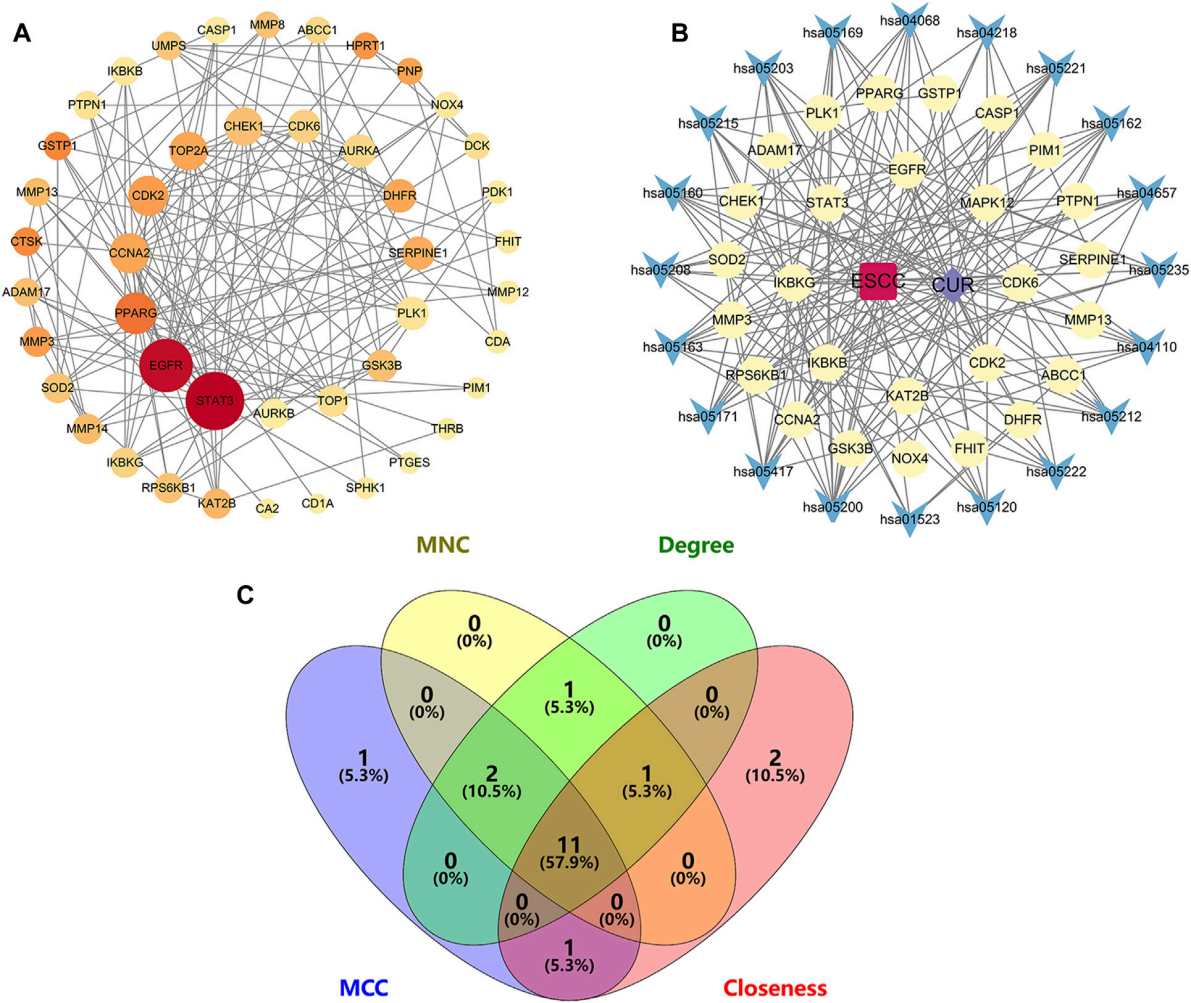
Network diagrams and core targets screening. **(A)** PPI network of potential targets for curcumin (CUR) therapy of esophageal squamous cell carcinoma (ESCC). **(B)** Drug-target-pathway network diagram. **(C)** Venn diagram showing the core targets screening by “cytohubba” of Cytoscape 3.9.1. Yellow circles are targets, blue triangles are pathways, purple diamond is CUR, and the red quadrant is ESCC.

To identify potential CUR targets for ESCC treatment, PPI networks were built for the 47 common targets ultilyzing STRING and Cytoscape v.3.9.1. Free targets were hidden during the screening, and 159 edges and 45 nodes in the PPI network were identified (Figure 4A). The average node degree was 6.77. The larger and darker nodes indicate higher degrees. Cytoscape 3.9.1 was utilized for constructing a drug-target-pathway network using the top 20 KEGG pathways (Figure 4B). The findings suggest that CUR possesses numerous targets and pathways that can be utilized for the treatment of ESCC. The top 15 targets were filtered using four key indicators in “cytohubba” of Cytoscape v.3.9.1. including the MCC, closeness, MNC, and degree. Eleven core targets were identified from the intersection of the four calculation methods (Figure 4C; Table 2).

**TABLE 2:** Top 15 targets information of PPI network.

	MCC		Degree		MNC		Clones	
Rank	Name	Score	Name	Score	Name	Score	Name	Score
1	CCNA2	5,468	STAT3	24	STAT3	23	STAT3	33.67
2	CHEK1	5,450	EGFR	21	EGFR	20	EGFR	32.17
2	TOP2A	5,450	PPARG	14	PPARG	14	PPARG	28.33
4	CDK2	5,322	CDK2	13	CDK2	13	CCNA2	27.42
5	PLK1	5,162	CCNA2	13	CCNA2	13	CDK2	27.25
6	AURKA	5,066	CHEK1	12	CHEK1	12	TOP2A	26.08
7	CDK6	5,050	TOP2A	12	TOP2A	12	CHEK1	25.75
8	AURKB	5,040	PLK1	9	PLK1	9	SERPINE1	25
9	TOP1	366	AURKA	9	AURKA	9	CDK6	24.75
10	DHFR	250	CDK6	9	CDK6	9	DHFR	24.42
11	EGFR	247	SERPINE1	9	SERPINE1	9	MMP14	24.33
12	STAT3	193	DHFR	9	DHFR	9	MMP3	24.17
13	PPARG	84	TOP1	8	TOP1	8	AURKA	24.08
14	MMP14	58	GSK3B	8	GSK3B	8	KAT2B	23.92
15	SERPINE1	56	MMP3	7	MMP3	7	IKBKG	23.92

### 3.4 The core targets of CUR in treating ESCC was validated by molecular docking

**FIGURE 5:**
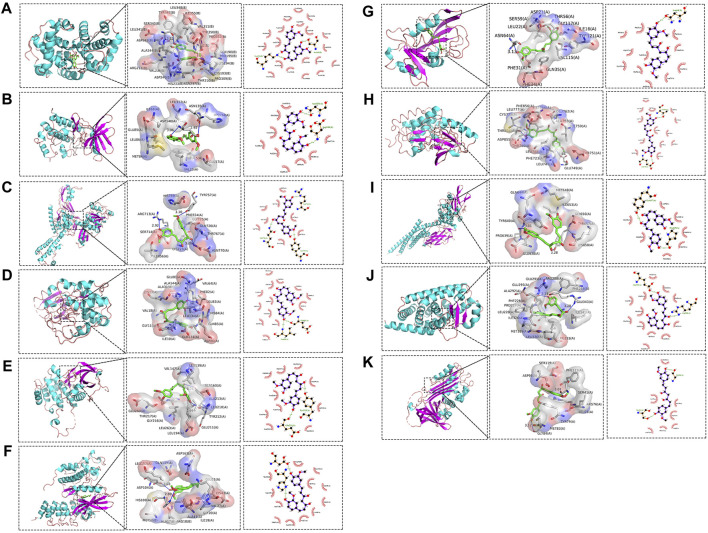
Molecular docking pattern of curcumin (CUR) and core target protein. **(A)**. CUR-CCNA2, **(B)** CUR-CHEK1, **(C)** CUR-TOP2A, **(D)** CUR-CDK2, **(E)** CUR-AURKA, **(F)** CUR-CDK6, **(G)** CUR-DHFR, **(H)** CUR-EGFR, **(I)** CUR-STAT3, **(J)** CUR-PPARG, and **(K)** CUR-SERPINE1.

Molecular docking was conducted on the 11 core targets that filtered by the PPI network. The core targets combination with CUR are shown in Figures 5A–K. Docking scores of CUR with 11 targets (CCNA2, CHEK1, TOP2A, CDK2, AURKA, CDK6, DHFR, EGFR, STAT3, and PPARG) were calculated. The docking scores were −4.7, −7.3, −6.3, −8.7, −7.1, −7.0, −9.2, −8.9, −5.9, −8.0 and −6.7 kcal/mol (Table 3), respectively. Stronger binding affinity is indicated by a lower docking score, whereas a score of < -5 kcal/mol indicated strong binding activity. This suggests that CUR can spontaneously bind to ten receptors (CHEK1, TOP2A, CDK2, AURKA, CDK6, DHFR, EGFR, STAT3, PPARG, and SERPINE1), similar to the equivalent crystallized ligands.

**TABLE 3:** Molecular docking data of curcumin (CUR) and target proteins.

Molecular name	Targets	PDB ID	Binding energy (kcal/Mol)
CUR	CCNA2	4eoj	−4.7
CUR	CHEK1	5oq5	−7.3
CUR	TOP2A	4fm9	−6.3
CUR	CDK2	6q4j	−8.7
CUR	AURKA	6vpm	−7.1
CUR	CDK6	1blx	−7.0
CUR	DHFR	1kmv	−9.2
CUR	EGFR	8a27	−8.9
CUR	STAT3	6njs	−5.9
CUR	PPARG	6ms7	−8.0
CUR	SERPINE1	7aqf	−6.7

### 3.5 Survival analysis of CHEK1 and CDK6 in ESCC

**FIGURE 6:**
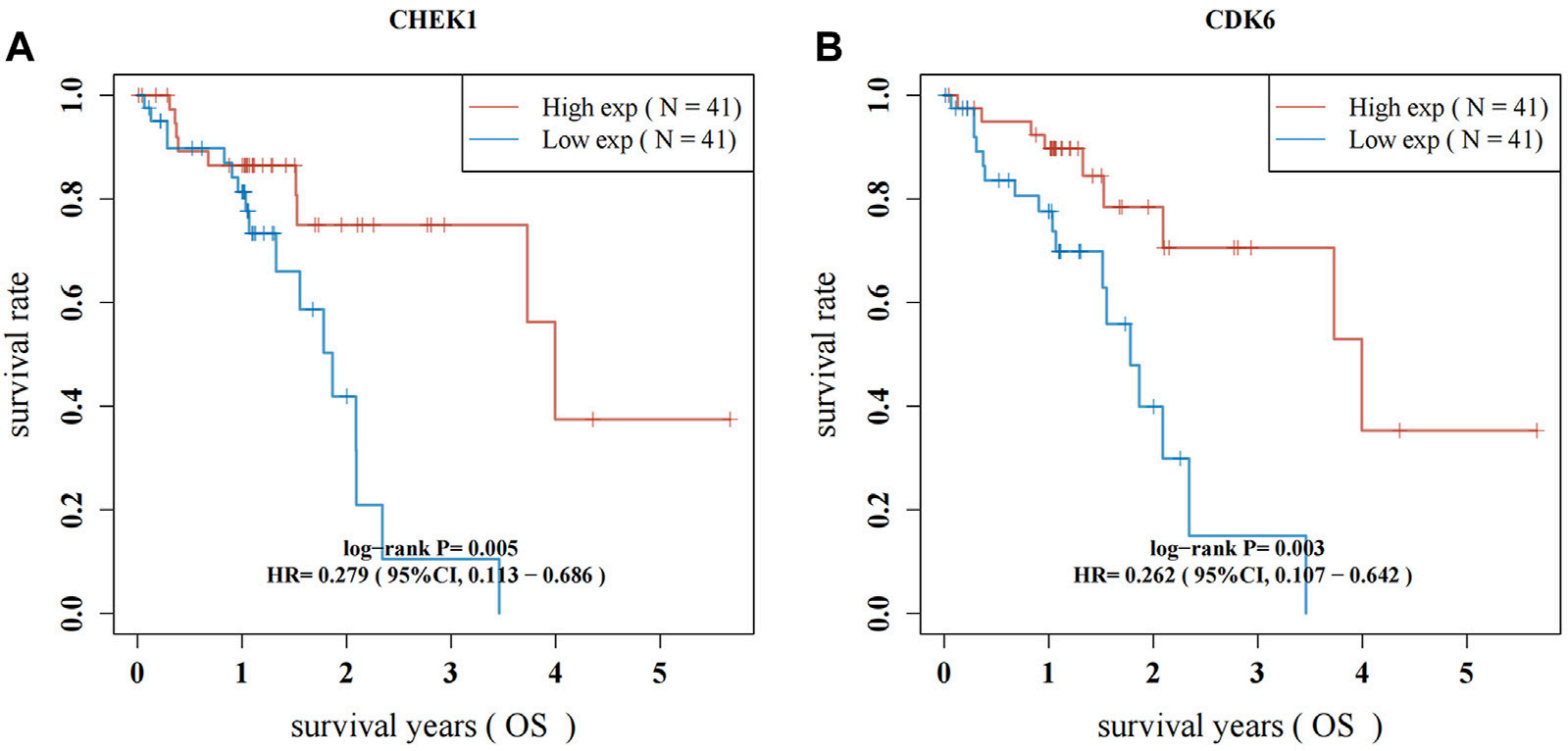
Survival analysis for hub genes of CHEK1 **(A)** and CDK6 **(B)** in ESCC. The red lines indicate high gene expression levels in sample groups, while green lines represent sample groups with low gene expression.

RNA-seq data from 82 ESCC patients and 11 normal para-carcinoma samples were obtained. Forty-seven hub genes from the PPI network and their clinical significance were identified by TCGA data (Supplementary Figure S1; Supplementary Table S1). Two genes, CHEK1 and CDK6, were associated with ESCC survival. ESCC patients who exhibited elevated levels of CHEK1 expression experienced a significantly shorter survival (*p* = 0.005; HR: 0.279, 95%CI:0.113–0.686). This result was similar to that for CDK6 (*p* = 0.003; HR:0.262, 95%CI:0.107–0.642) (Figures 6A, B).

### 3.6 Experimental verification of the function of CUR in ESCC cells

The treatment of ESCC has widely recognized the therapeutic potential of focusing on the cell cycle (Wang et al., 2017; Zhang et al., 2023) and cell senescence (Chen and Pan, 2017). According to the enrichment results from GO and KEGG, it was found that CUR could potentially disrupt the apoptosis pathways, the G2/M transition in the cell cycle, and cellular senescence.

**FIGURE 7:**
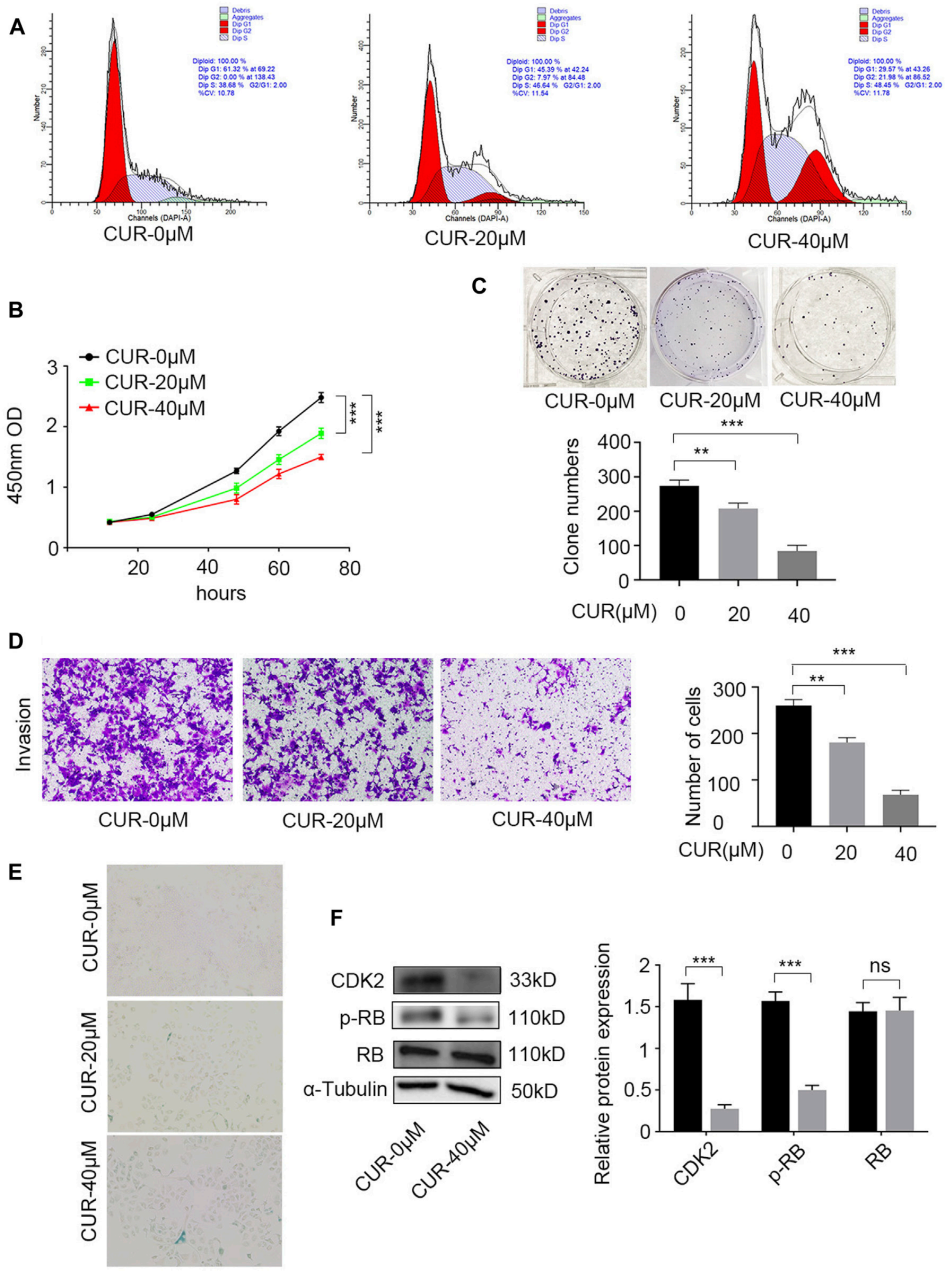
Experimental verification the function of curcumin (CUR) in KYSE-140 cells. **(A)** Cell cycle analysis by PI staining and flow cytometry. **(B,C)** Cell Counting Kit-8 (CCK-8) experiment and colony formation assay were utilized to identify cell proliferation ability of KYSE-140 cells. **(D)** The effects of CUR in KYSE-140 cell invasion ability were measured by Transwell assays. **(E)** The effects of CUR in KYSE-140 cell senescence were measured by SA-β-gal staining. **(F)** Western blot of CDK2, RB, and p-RB expression in KYSE-140 cells treated with CUR. *p < 0.05, **p < 0.01, ***p < 0.001.

Further experiments were conducted to validate this hypothesis. The cell cycle was identified using flow cytometry and PI staining. After 48 h of treatment, CUR enhanced the cell count in the G2/M and S phases. At 40 μΜ, 48.45% of KYSE-140 cells showed arrest of the S-phase cell cycle (Figure 7A).

We examined the effects of CUR on KYSE-140 proliferation using CCK8 and colony formation assays. CCK-8 assays indicated that compared to control cells (0 μΜ CUR), KYSE-140 cell line treated with CUR with two different doses (20 and 40 μΜ) both exhibited significantly decreased proliferation ability (*p* < 0.001) (Figure 7B). Similarly, colony formation was significantly decreased in KYSE140 treated with 20 μΜ CUR (*p* < 0.01) and 40 μΜ CUR (*p* < 0.001) compared to control cells (0 μΜ CUR) (Figure 7C). Next, we investigated whether CUR inhibited cell invasion in ESCC cell lines using Transwell invasion assays. The results showed, compared to the rates in control cells (0 μΜ CUR), the invasion rates decreased significantly in KYS140 cell lines treated with 20 μΜ CUR (30.57% reduction, *p* < 0.01) and 40 μΜ CUR (30.57% reduction, *p* < 0.001), respectively (Figure 7D). KEGG enrichment analysis also indicated that cellular senescence is one of the most important pathways of CUR in treating ESCC. We reperformed the experiments and acquired the image data of treated cells (SA-β-gal measurement). After CUR treatment, KYSE-140 cell markedly enlarged (Figure 7E) and senescent cells also clearly increased (blue color). Among the top eleven potential targets of CUR, the docking of CDK2 had a lower binding score (−8.7 kcal/mol). Lower docking scores indicate stronger binding affinity. After CUR treatment (40 μΜ), the level CDK2 (*p* < 0.001) and p-RB (*p* < 0.001) were both significantly decreased, while total RB expression did not change significantly (Figure 7F).

## 4 Discussion

The mortality rate of ESCC is high and poses a threat to human health. CUR has been widely used in cancer treatment (Bar-Sela et al., 2010; Zoi et al., 2021). A recent study found that CUR has the ability to regulate the circNRIP1/miR-532-3p/AKT pathway, resulting in the suppression of ESCC (Luo et al., 2023). Furthermore, CUR induces apoptosis (Liu et al., 2018; Deng et al., 2021) and suppresses ESCC cell proliferation (Hosseini et al., 2018), and blocks the growth of primary ESCC-derived xenografts *in vitro* (Liu et al., 2018). However, there is a dearth of research on the anti-cancer mechanisms of CUR in ESCC. Network pharmacology is a powerful tool based on network biology, traditional pharmacology, chemoinformatics, and bioinformatics for studying the complex mechanisms of TCM (Zhu et al., 2021). This study aimed to investigate the mechanisms and molecular targets of CUR in ESCC using network pharmacology, database mining, and experimental validation to provide novel ideas for the comprehensive treatment of ESCC.

In the present study, we identified 81 potential CUR targets in ESCC by examining the intersection of ESCC and CUR related targets. The 81 potential targets were extracted from TCGA dataset and 47 differentially expressed genes were identified in ESCC samples. To illustrate the anti-ESCC mechanism of CUR, DAVID was used to enrich the GO and KEGG pathways for 47 potential targets. Subsequently, the PPI network of the drug-target-pathway network was constructed and analyzed, and 11 core targets were identified. Furthermore, the 11 core targets were verified by molecular docking studies, and the results suggested that CUR may spontaneously bind to ten receptors. Additionally, survival analysis was performed on the basis of TCGA data sets. Among these potential targets, CHEK1 and CDK6 were associated with greater survival. To confirm the impact of CUR on ESCC cells, *in vitro* experiments were ultimately conducted.

The GO enrichment results showed that CUR regulated apoptosis and the progression from G2 to M phase in the mitotic cell cycle in ESCC cells. Apoptosis, which is a specific form of programmed cell death, mediated by both intrinsic and extrinsic pathways (Singh and Lim, 2022). Apoptotic mechanisms are critical in cancer treatment, and many therapeutic strategies target these mechanisms (Carneiro and El-Deiry, 2020). Targeting the apoptotic machinery is particularly important (Hong et al., 2020; Zhang et al., 2023). Multiple pathways have been demonstrated in numerous studies to cause cancer cell apoptosis by CUR. Apoptosis and cell cycle regulation are intricately associated. DNA damage and cell cycle can lead to irreversible cell cycle arrest, which induces cell apoptosis. Researchers have shown that regulation of key cell cycle proteins can induce cell cycle arrest in ESCC cells and produce anti-tumor effects (Yao et al., 2015; Kwak et al., 2020). Prior research has shown that CUR induces cell cycle arrest in cells of acute myeloid leukemia (Zhou et al., 2021), colorectal cancer cells (Li et al., 2021), and pancreatic cancer cells (Zhu and Bu, 2017). In this study, we observed that apoptosis and the G2/M transition of the mitotic cell cycle are the two main GO-enriched processes through which CUR regulates the treatment of ESCC.

Based on the findings of KEGG enrichment analysis, CUR has the potential to regulate various signaling pathways including the FoxO pathway, cell cycle, cellular senescence, IL-17 pathway, and several pathways associated with cancer. The FoxO signaling pathway has been identified as a promising candidate for cancer therapy. The interleukin 17 (IL-17) family of cytokines have a strong impact on both acute and chronic inflammation. However, reports on the FoxO and IL-17 signaling pathways in ESCC remain limited. Cellular senescence is related to the cellular cycle because it is a highly stable cycle arrest caused by a range of stresses. Induction of cell cycle arrest (Kwak et al., 2021) and cellular senescence (Chen et al., 2023) an essential approach to combat cancer in ESCC patients. To better understand the pathways regulated by CUR in the treatment of ESCC, two signaling pathways were mapped and relevant targets were marked.

By utilizing the TCGA dataset, the intersection and filtration of the CUR and ESCC targets yielded a total of 47 potential targets for ESCC treatment. The drug-target pathway network analysis revealed that CUR activates multi-target and multiple pathways in the treatment of ESCC. A PPI network was utilized to choose the top 11 primary objectives. The analysis of molecular docking indicated that CUR binds to 10 predicted core targets (CHEK1, TOP2A, CDK2, AURKA, CDK6, DHFR, EGFR, STAT3, PPARG, and SERPINE1). Survival analysis using TCGA data revealed that CHEK1 and CDK6 were positively correlated with ESCC survival. The serine/threonine-specific protein kinase CHEK1 can regulate cell cycle arrest and coordinates DNA repair (Goto et al., 2012). Moreover, the presence of CHEK1 correlates with the severity and reappearance of tumors, suggesting its role in tumor progression (Fadaka et al., 2020). In ESCC patients, Li et al. discovered a significant association between CHEK1 genetic variations and both overall survival (OS) and disease-free survival (DFS) (Li et al., 2016). Similarly, we found a positive correlation between CHEK1 and OS of patients with ESCC in the TCGA data set. CDK6 mediates the cellular transition to the S phase and has a vital function in the onset, development, and persistence of various forms of cancer (Goel et al., 2022). CDK6 amplification is significantly correlated with tumor size and indicates a better prognosis for ESCC (Liu et al., 2023). Our findings align with this outcome.

Of the other CUR core targets, TOP2A, an isoform of the DNA topoisomerase II (TOP2) enzyme, plays an essential role in cell division, dendritic cell division, chromosome condensation, and segregation during mitosis (Uusküla-Reimand and Wilson, 2022). The TOP2 enzyme is routinely targeted in chemotherapy to treat solid tumors and hematological malignancies (Vann et al., 2021). Cyclin-dependent kinase 2 (CDK2) plays a crucial role in controlling the cell cycle and various biological functions (Tadesse et al., 2020). In a subset of tumors, CDK2 inhibition elicits anti-tumor activity by deregulating its regulatory subunits (Tadesse et al., 2020). Both *in vitro* and *in vivo* experiments indicated that reduced expression of CDK2 pathway suppresses ESCC growth (Zhou et al., 2023). AURKA demonstrates considerably elevated levels of expression in various cancer tissues compared to normal control tissues (Du et al., 2021). Bao et al. found that by targeting AURKA, oxethazaine inhibits the proliferation and metastasis of ESCC *in vitro* and *in vivo* (Bao et al., 2022). For the treatment of cancer, the targeting of dihydrofolate reductase (DHFR) enzymes has been acknowledged for a considerable time (Raimondi et al., 2019). The EGFR, which is responsible for transmitting significant signals of growth factors from the external environment to cells via cytoplasmic kinase activity (Passaro et al., 2021), shows a connection with unfavorable prognosis of ESCC when it is excessively expressed (Jiang et al., 2015). STAT3 functions as a crossroad for multiple cancer-causing signaling pathways and controls the immune reaction to tumors (Zou et al., 2020). The nuclear receptor PPARG is closely associated with the clinical outcome of patients who have undergone surgery for ESCC (Sun et al., 2015). SERPINE1, an inhibitor of tissue plasminogen activator and urokinase, plays a role in enhancing tumor advancement and spreading (Chen et al., 2022).

According to previous research, CUR has the ability to trigger apoptosis and halt the cell cycle in the S phase. As a result, it effectively hinders the migration and growth of cells associated with endometrial carcinoma (Zhang et al., 2019). Several researches have validated that CUR triggers apoptosis in ESCC cells by inhibiting the STAT3 pathway (Liu et al., 2018; Wang et al., 2018). However, the exact way in which CUR works against ESCC is still not understood. Based on the findings from the GO and KEGG analyses, CUR has the potential to address ESCC by modulating pathways related to cell cycle, programmed cell death, and cellular aging. Our experiments showed that CUR induced a pause in the cell cycle at the G2/M and S stages. CCK8 experiments and clone formation assays indicated that CUR significantly inhibited ESCC cell proliferation. Furthermore, Transwell invasion assays demonstrated that CUR inhibited ESCC cell invasion and SA-β-gal measurement indicated that CUR can induce ESCC cell senescence. Western blot results showed that CUR may treat ESCC by inhibiting CDK2/RB pathway.

## 5 Conclusion

In summary, our findings from network pharmacology, molecular docking, and *in vitro* experimental confirm that CUR possesses the capacity to inhibit the proliferation and invasion of ESCC cells. This is achieved through the induction of cell cycle arrest, regulation of FoxO, cell senescence, IL-17, and many cancer-related signaling pathways. CUR may exert anti-ESCC effects by interacting with CHEK1, TOP2A, CDK2, AURKA, CDK6, DHFR, EGFR, STAT3, PPARG, and SERPINE1. Nevertheless, further investigations are required to guarantee the dependability of the results. The findings of this research offer a fresh basis for additional exploration and empirical verification of the significance of CUR in managing ESCC.

## Data Availability

The original contributions presented in the study are included in the article/Supplementary Material, further inquiries can be directed to the corresponding authors.

## Abbreviations

CUR, curcumin; EC, esophageal cancer; ESCC, esophageal squamous cell carcinoma; GO, Gene Ontology; KEGG, Kyoto Encyclopedia of Genes and Genomes; MCC, the maximum clique centralities; MNC, maximum neighborhood components; PPI, protein–protein interaction; TCM, traditional Chinese medicines.
