# Supplementary material for: Network pharmacology and molecular docking reveal the mechanisms of curcumin activity against esophageal squamous cell carcinoma
Source: Front Pharmacol. 2024 Apr 3;15:1282361. doi: 10.3389/fphar.2024.1282361 (PMC11021710; doi:10.3389/fphar.2024.1282361)

**Supplementary Figure 1** Forest plot. The *P*-values, risk coefficients (HR), and univariate analysis of the expression and prognostic characteristics of the core targets from the single factor cox analysis of genes in ESCC.


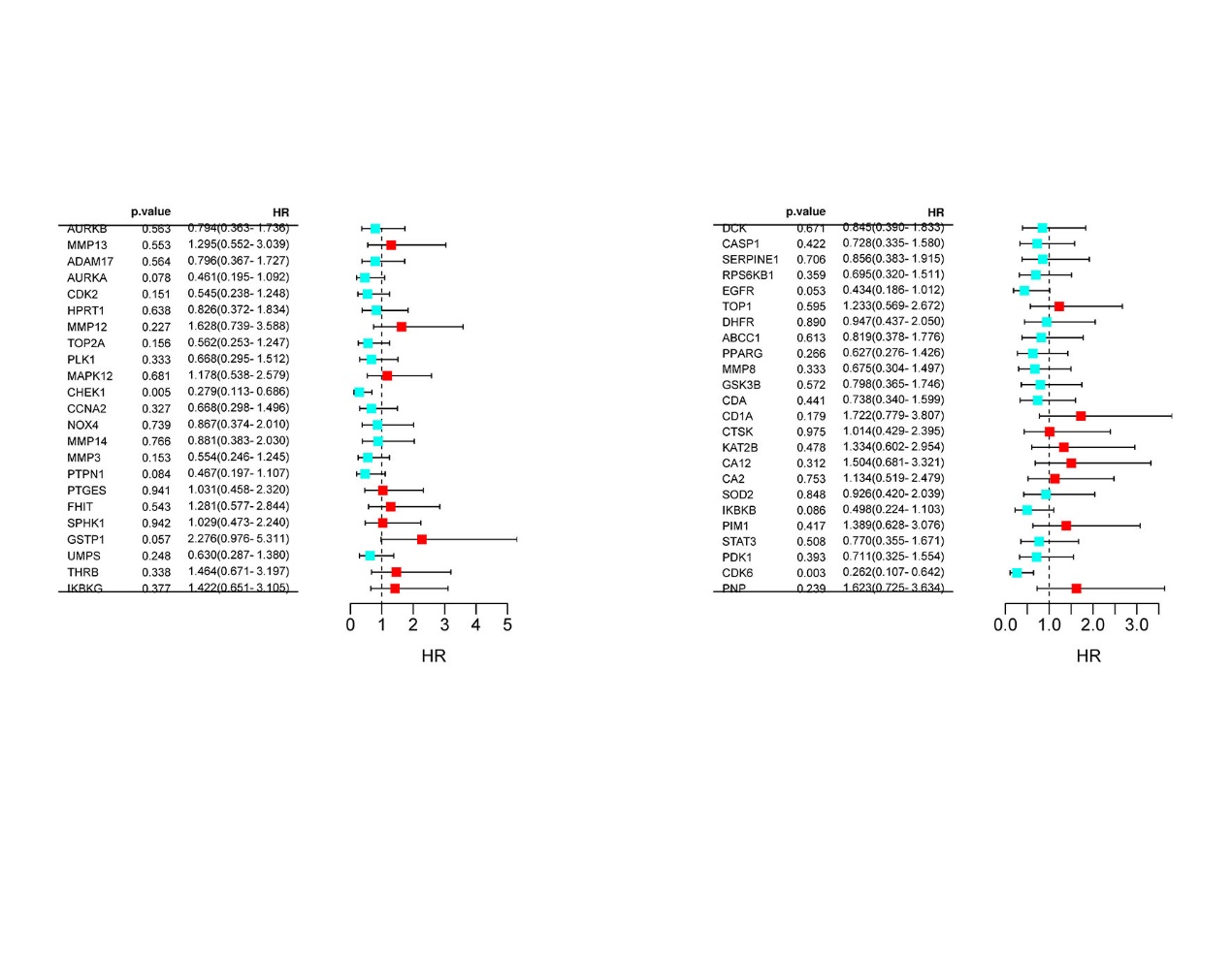

Supplement: Supplementary file 2 [file DataSheet1.docx]
